# Recent acquisition of imprinting at the rodent *Sfmbt2 *locus correlates with insertion of a large block of miRNAs

**DOI:** 10.1186/1471-2164-12-204

**Published:** 2011-04-21

**Authors:** Qianwei Wang, Jacqueline Chow, Jenny Hong, Anne Ferguson Smith, Carol Moreno, Peter Seaby, Paul Vrana, Kamelia Miri, Joon Tak, Eu Ddeum Chung, Gabriela Mastromonaco, Isabella Caniggia, Susannah Varmuza

**Affiliations:** 1 Department of Cell and Systems Biology, University of Toronto, 25 Harbord St., Toronto, Ontario, M5S 3G5, Canada; 2 Department of Physiology, Development and Neuroscience, University of Cambridge, Downing Street, Cambridge, CB2 3EG, UK; 3 Department of Physiology, Medical College of Wisconsin, 8701 Watertown Plank Road Milwaukee, WI, USA, 53226, USA; 4 Department of Biomedical Sciences, University of Guelph, 50 Stone Rd. E.,Guelph, Ontario, N1G 2W1, Canada; 5 Peromyscus Genetic Stock Center & Department of Biological Sciences, University of South Carolina, Columbia, SC 29208, USA; 6 Reproductive Physiology, Toronto Zoo, 361A Old Finch Avenue, Toronto, Ontario, M1B 5K7, Canada; 7 Samuel Lunenfeld Research Institute, Mount Sinai Hospital, Joseph and Wolf Lebovic Health Complex, 600 University Avenue, Toronto, Ontario, M5G 1X5, Canada

## Abstract

**Background:**

The proximal region of murine Chr 2 has long been known to harbour one or more imprinted genes from classic genetic studies involving reciprocal translocations. No imprinted gene had been identified from this region until our study demonstrated that the PcG gene *Sfmbt2 *is expressed from the paternally inherited allele in early embryos and extraembryonic tissues. Imprinted genes generally reside in clusters near elements termed Imprinting Control Regions (ICRs), suggesting that *Sfmbt2 *might represent an anchor for a new imprinted domain.

**Results:**

We analyzed allelic expression of approximately 20 genes within a 3.9 Mb domain and found that *Sfmbt2 *and an overlapping non-coding antisense transcript are the only imprinted genes in this region. These transcripts represent a very narrow imprinted gene locus. We also demonstrate that rat *Sfmbt2 *is imprinted in extraembryonic tissues. An interesting feature of both mouse and rat *Sfmbt2 *genes is the presence of a large block of miRNAs in intron 10. Other mammals, including the bovine, lack this block of miRNAs. Consistent with this association, we show that human and bovine *Sfmbt2 *are biallelic. Other evidence indicates that pig *Sfmbt2 *is also not imprinted. Further strengthening the argument for recent evolution of *Sfmbt2 *is our demonstration that a more distant muroid rodent, *Peromyscus *also lacks imprinting and the block of miRNAs.

**Conclusions:**

These observations are consistent with the hypothesis that the block of miRNAs are driving imprinting at this locus. Our results are discussed in the context of ncRNAs at other imprinted loci.

Accession numbers for *Peromyscus *cDNA and intron 10 genomic DNA are [Genbank:HQ416417 and Genbank:HQ416418], respectively.

## Background

Genomic imprinting is an epigenetic process that affects a small subset of genes resulting in their expression/repression in a parent of origin dependent fashion. One set of imprinted genes is expressed only from the paternally inherited allele, while another set is expressed only from the maternally inherited chromosome. The imprint is reset at each generation when the two haploid genomes are separate, either during gametogenesis or immediately after fertilization, when the two genomes are physically separated in their own pronuclei.

Imprinted genes generally reside in clusters around a cis acting element called an Imprinting Control Region (ICR) that exerts its effects over a large chromosomal domain (up to 4 Mb) (reviewed in [1]). Imprinted domains can contain genes that are biallelic, paternally expressed or maternally expressed. Monoallelic expression can be universal (ie in all tissues tested), or limited to a subset of tissues. The most common type of tissue specific limitation is to the extraembryonic lineages, exemplified by the placenta and the yolk sac. This latter observation has provided strong support for the idea that the evolutionary origins of imprinting are rooted in extraembryonic tissue biology.

In mouse uniparental embryos, the tissue most severely affected is the trophoblast, the precursor of several placental cell types. Gynogenetic/parthenogenetic embryos have almost none by midgestation, whereas androgenetic embryos have hyperplastic trophoblast. Moreover, derivation of trophoblast stem cells from parthenogenetic mouse blastocysts is extremely inefficient, and is accompanied by selective loss of imprinting of several genes (Miri and Varmuza, Parthenogenetic embryos are impaired in their ability to make TS cells, manuscript in preparation). These observations led us to hypothesize that a gene critical for trophoblast establishment/function in blastocysts is expressed from a paternally-inherited chromosome, and is therefore missing from gynogenetic/parthenogenetic embryos. A microarray comparison of the transcriptomes of androgenetic and gynogenetic blastocysts revealed that the PcG gene *Sfmbt2 *is expressed almost exclusively from the paternal allele starting at the blastocyst stage [2]. Monoallelic expression in all tissues is preserved up to e7.5, after which a high level monoallelic expression is preserved in extraembryonic tissues, while significantly reduced, but biallelic, expression in somatic tissues can be observed. These observations place *Sfmbt2 *in the class of imprinted genes that are specific to the extraembryonic tissues.

*Sfmbt2 *maps to the proximal region of Chromosome 2. This region was mapped as imprinted through the chromosome translocation studies of Cattanach and colleagues [3,4], but no imprinted gene had been identified until our study [2]. Here we extend our analysis of the domain surrounding *Sfmbt2*, and the search for a potential ICR. Our results indicate that *Sfmbt2 *is the only gene within a 4 Mb region that is imprinted, and that we find no evidence of a classical ICR displaying robust germline specific DNA methylation that is preserved after fertilization. We also show that imprinting appears to be unique to mice and rats, and is associated with the acquisition of a block of miRNAs in one of the introns. Our results suggest that we have caught a gene "in the act" of becoming imprinted.

## Results

### *Sfmbt2* is the only imprinted gene in this domain

The largest imprinted cluster, the *Snrpn *locus, is 3.7 Mb. We therefore assessed the allelic expression of 23 annotated protein coding genes, including *Sfmbt2*, within a 4.3 Mb domain, from *Gata3 *to *Mrc1*. Several unannotated genes were also tested for allelic expression, but these will not be discussed further. *Gata3 *lies 500 kb proximal to *Sfmbt2*; the next most proximal gene, *Cugbp2*, is 3.3 Mb upstream of *Gata3*, indicating the presence of a gene desert. *Prkcq*, immediately telomeric to *Sfmbt2*, is not expressed in extraembryonic tissues (data not shown).

**Figure 1:**
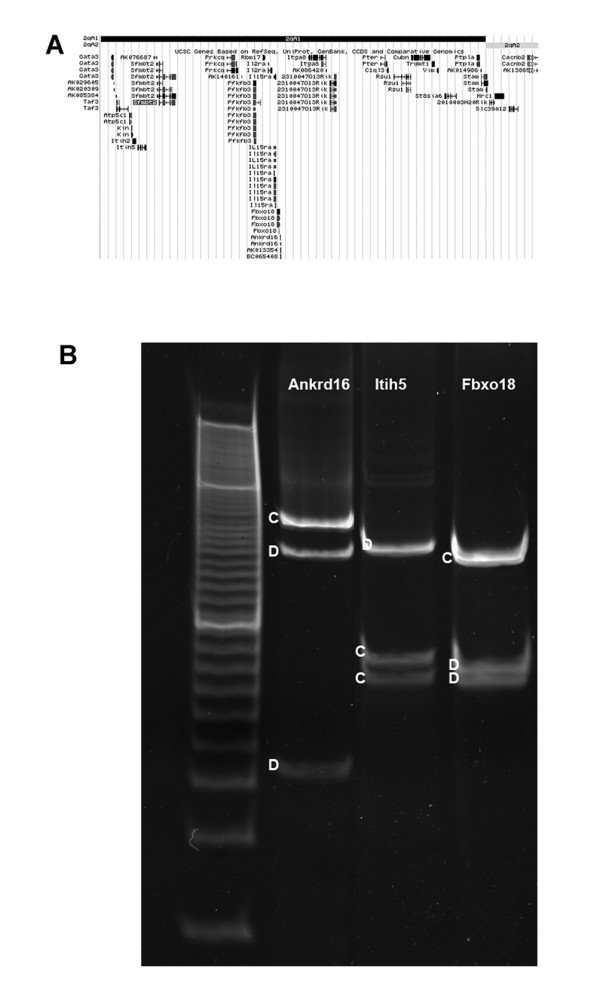
**Representative SNP Analysis of *Sfmbt2 *Domain Genes**. A. Twenty two genes flanking *Sfmbt2* were analysed for allelic expression in placenta or yolk sac from C57BL6 (D) X Castaneus (C) mid gestation fetuses. Illustrated is a screen shot of the UCSC genome browser image of the region encomapssing *Gata3 *to *Mrc1 *on Chromosome 2. *Prkcq *is not expressed in extraembryonic tissues. B. Shown are three representative genes, *Ankrd16, Itih5 *and *Fbxo18*, analysed by allele-specific expression RFLP. The two parental alleles are clearly present in placenta cDNA for all three genes. Results for the other genes can be seen in Additional file 1, Figure S1.

With the exception of *Sfmbt2*, none of the genes assayed displayed monoallelic expression in *Domesticus X Castaneus *or *Castaneus X Domesticus *placenta or yolk sac (Figure 1 and Additional file 1, Figure S1). Interestingly, a recent study of e9.5 hybrid embryos, using a massively parallel sequencing approach, revealed several new imprinted genes. However, none were found in the proximal region of Chromosome 2, including *Sfmbt2 *[5]. This is consistent with our observation that *Sfmbt2 *becomes biallelic in embryo-derived somatic tissues. None of the neighbouring genes is imprinted in embryo. Together with our findings, these results indicate that *Sfmbt2 *is the only imprinted gene within a 4.3 Mb region of proximal Chromosome 2, and only in early embryo and extraembryonic tissues.

**Figure 2:**
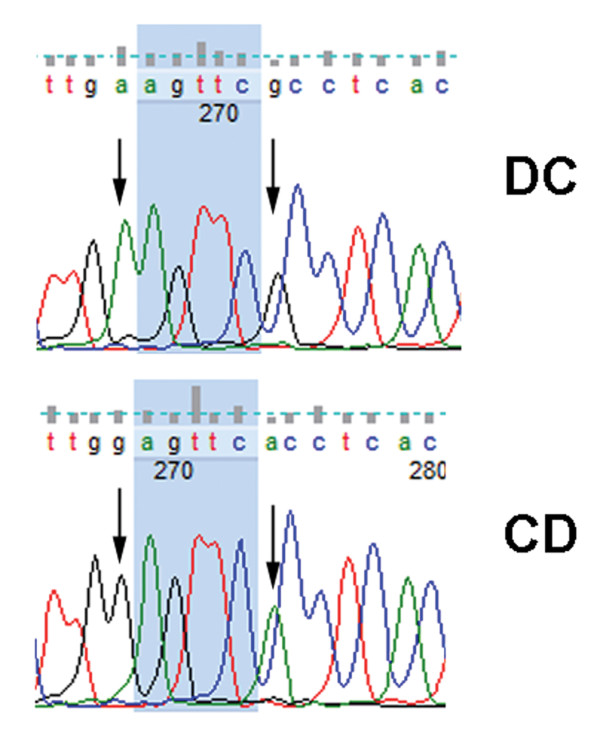
**A non-coding antisense transcript is expressed from the paternal allele**. Several annotated SNPs in the predicted non-coding antisense transcript that starts in the first common intron were located in the downstream exons. cDNAs from *Domesticus X Castaneus *(DC) and the reciprocal (CD) placentae were sequenced and found to contain only paternal alleles. Five polymorphisms were observed, two of which are illustrated here. None of the exons in the antisense transcript overlap exons in processed *Sfmbt2 *transcripts.

*Sfmbt2 *has multiple transcriptional starts, stops and differential splicing (see Ensembl or UCSC database). The two transcriptional starts sites (TSS) are utilized differently; the more 5' start is specific to extraembryonic tissues, while the 3' start is ubiquitous [2]. There is a non-coding antisense transcript that starts in the first common intron downstream of the two *Sfmbt2 *TSS. The putative promoter for this antisense transcript embodies part of a CpG island (described below). In placenta, this transcript is also imprinted and like *Sfmbt2 *is expressed from the paternal allele (Figure 2). This result is in contrast with other imprinted gene domains that possess antisense non-coding transcripts, for example the *Kcnq1ot1 *and *Airn *transcripts in the *Kcnq1 *and *Igf2r *loci, respectively (reviewed in [6]). In these latter cases, the antisense transcript is expressed from the opposite allele as the sense transcript.

### Oocyte DMR is Sparse and Largely Asymmetric

Fifteen imprinted domains are regulated by germline Differentially Methylated Regions (DMRs); twelve are methylated on the maternal chromosome in somatic tissues and in oocytes, while three are methylated on the paternal allele in somatic tissues and in sperm [7]. Several other DMRs have been identified, but not tested for ICR function. In addition, many imprinted genes display a characteristic chromatin signature [8]. A search of the Broad Institute ChIP-seq database revealed strong peaks of H3K4Me3 and H3K27Me3 binding across the *Sfmbt2 *sense and antisense TSS and coinciding with a CpG island; however, in contrast with other germline DMRs, there was no discernible H3K9Me3 or H4K20Me3 binding in ES cells [9].

**Figure 3:**
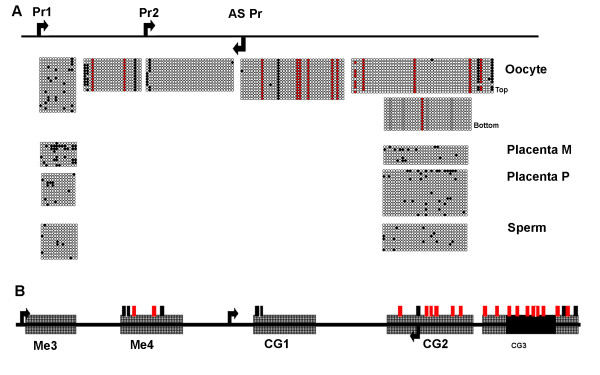
**Methylation of *Sfmbt2 *CpG Island**. A. Five amplicons representing 140 CpG sites across the transcriptional start sites for both the sense and antisense transcripts at the *Sfmbt2 *locus were queried for methylation in sperm and oocyte genomic DNA by bisulfite mutagenesis. Two amplicons, at the 5' and 3' extremes of the CpG island, Me3 and CG3, were also examined in C57BL6 X Castaneus F1 placenta genomic DNA to determine parental allele methylation. Black circles represent CpGs that survived bisulfite conversion; open circles represent CpGs that were converted to TG; red circles represent asymmetric cytosines in oocyte genomic DNA that survived conversion in most clones; grey circles represent cytosines in a CpG context that were methylated on only the bottom strand in oocyte genomic DNA. Each line represents a unique clone; duplicate clones were not scored. Bisulfite conversion was approximately 95%. The apparent two CpG sites in CG1 are only separated by a single base pair, and are therefore assumed to be a single site. B. Relative location of bisulfite amplicons within CpG island. Hatched boxes represent amplicons for which only one strand was queried; solid box (CG3) was analysed on both the top and bottom strands. Hash marks indicate the relative location of the methylated cytosines in oocyte genomic DNA; black = CpG, red = asymmetric. Of 25 methylated cytosines, 18 were confirmed to be asymmetric, including the four CpG sites in CG3 illustrated in panel A.

There is a large CpG cluster of approximately 5.3 kb that spans the TSS for *Sfmbt2*, including the start site that is used only in extraembryonic tissues (herein called Promoter 1), and extends 3 kb downstream of the ubiquitous TSS (herein called Promoter 2). The TSS for the non coding (nc) antisense RNA also resides within the CpG island. Five amplicons from this region were queried for methylation by bisulfite mutagenesis in oocyte and sperm genomic DNA (Figure 3A). Two of the five amplicons, Me3 and CG3, contained polymorphisms that allowed us to distinguish parental alleles after bisulite mutagenesis and sequencing; these two amplicons were analysed in placental DNA from C57BL6 X *Castaneus *F1 placentae. Unlike other DMRs from well conserved imprinted domains, the CpG cluster spanning the promoter region of *Sfmbt2 *did not contain a block of methylated CpGs; methylated cytosines were distributed sparsely throughout the CpG cluster (Figure 3B). The amplicon spanning the Promoter 1 start site (Me3) is not noticeably methylated in either sperm or oocyte, although there is a slight increase in methylation on the maternal allele in both oocyte and placenta. Methylation of Me3 in fetal brain is similarly sparse, and does not display any allelic bias (not shown). Four of the amplicons contained CpG sites that were methylated in oocyte but not sperm; however, in two cases this observation was confined to a single site, and in one case to four out of 40 potential sites. Interestingly, three of these four amplicons contained methylated asymmetric cytosines in oocyte DNA, a conclusion that was drawn from the fact that all unique clones contained Cs at these positions. Following this observation, the other strand of CG3 was subjected to bisulfite mutagenesis and sequencing to assess the symmetry of the four CpG sites identified in the initial analysis. All four proved to be asymmetrically methylated, and an additional five asymmetric sites were identified. This observation strongly suggests that DNA methylation may be a secondary consequence of ordered chromatin structure in oocytes. None of the maternal methylated cytosines in CG3 was conserved in placental DNA.

### Rat *Sfmbt2* is Imprinted, but Bovine *Sfmbt2* is Biallelic

**Figure 4:**
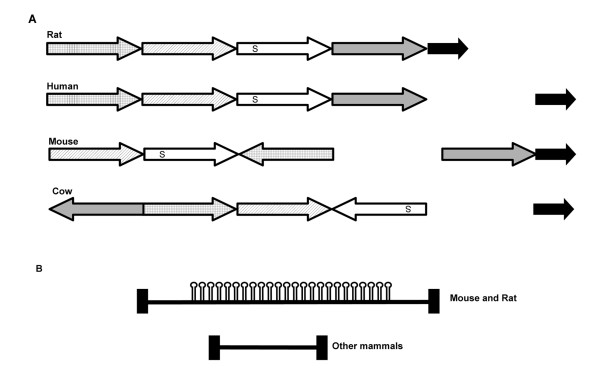
**Genomic organization of Mammalian *Sfmbt2 *Genes**. A. Diagram illustrating the syntenic organization of the *Sfmbt2 *domain from four mammals - rat, mouse, human and cow - with different blocks of genes represented by arrows. The location of *Sfmbt2 *is indicated with "S". The rat domain may be ancestral. B. Intron 10 in mice and rats possesses a large block of miRNAs, while other mammalian genes do not.

The human *Sfmbt2 *gene is not imprinted (Additional file 2, Figure S2). In examining the genomic sequences within and near mouse and human genes, it became apparent that the mouse gene differs from the human gene in two respects:

1. The syntenic region of the mouse and human domains is quite different. A comparison of human, mouse, rat and cow syntenic regions indicates that the rat domain is likely ancestral; the human gene has undergone one small translocation; the mouse and bovine domains are rearranged significantly (Figure 4A).

2. Rat and mouse genes possess a large block of miRNAs in intron 10, while all other mammals with well annotated genomes, including human and bovine, do not have this block of miRNAs (Figure 4B and Additional files 3 and 4, Figures S3 and S4). The miRNAs fall within the mir-467 family.

These observations raised the possibility of testing whether chromosomal arrangements are important for acquisition of the imprint in the mouse gene, or whether the block of miRNAs is the critical feature driving imprinting at this locus. If the rat gene is biallelic, then some aspect of the chromosomal rearrangement in the mouse may have led to the acquisition of monoallelic expression through some kind of position effect. On the other hand, if the rat gene is imprinted, then recent insertion of the block of miRNAs may play a pivotal role in the establishment of a new imprinted domain.

**Figure 5:**
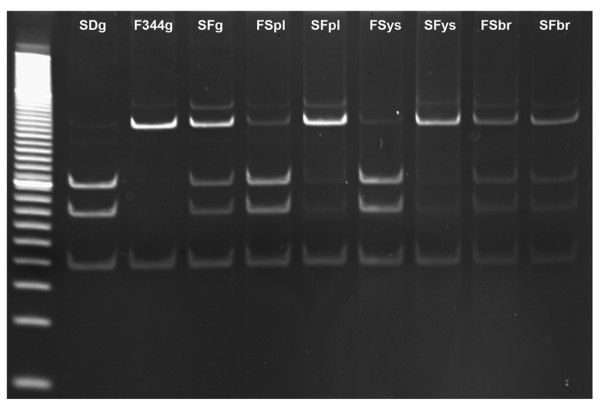
**Rat *Sfmbt2 *is Imprinted in Extraembryonic Tissues**. SNPs were identified in the 3' UTR of the rat *Sfmbt2 *gene in Sprague Dawley (SD) and F344 (F) strains. Midgestation placentas, yolk sacs and brains from crosses of SD females with F344 males (SF) and the reciprocal (FS) were analysed for allelic expression using polymorphic AciI sites. While genomic DNA and brain cDNA displayed clear biallelic patterns, both yolk sac and placenta cDNA contained mainly paternal alleles.

We found a polymorphism in the 3' UTR of the rat *Sfmbt2 *gene in F344 and Sprague Dawley rats, and assayed allelic expression in mid-gestation placenta, yolk sac and brain from (F344 X SD) F1 and (SD X F344) F1 fetuses. As with mice, placenta and yolk sac expressed predominantly the paternal allele, while brain showed clear biallelic expression (Figure 5).

**Figure 6:**
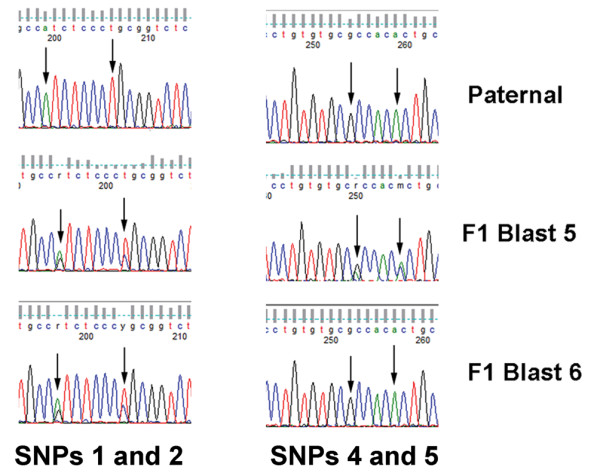
**Bovine *Sfmbt2 *is Biallelic**. Several SNPs in the 3' UTR of the bovine *Sfmbt2 *gene were found to be biallelic in two pools of 5 blastocysts each. The sperm used for fertilizing all of the oocytes was from the same male Plains bison. The second set of SNPs (right panel) is uninformative in pool 6.

The bovine genomic region encompassing *Sfmbt2 *has undergone significant rearrangements in comparison with rat, mouse and human (Figure 4A). Polymorphisms between two species of cattle, *Bos taurus *and plains bison, were used to assess allelic expression in F1 blastocysts. Unlike mouse blastocysts, which display monoalleic expression from the paternal allele, bovine blastocysts are clearly biallelic (Figure 6). Thus, chromosomal rearrangements are not associated with the acquisition of imprinting, at least not in cattle.

### Peromyscus *Sfmbt2* Lacks a Large Block of miRNAs and is Biallelic

**Figure 7:**
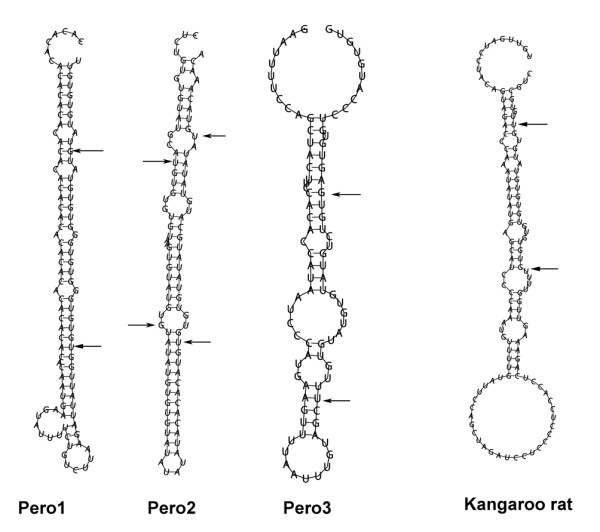
**Peromyscus and Kangaroo Rat Putative pre-miRNAs in Intron 10**. Intron 10 sequence was BLAST searched against the mirBase database. Sequences that displayed homology with mir-467 family members were then subjected to folding with RNAFold to assess the liklihood of correct processing by Drosha and Dicer. Alignment of the putative pre-miRNAs with known mir-467 family members revealed the position of processed miRNAs in relation to the stem-loop (arrows). The Pero1 and Pero2 sequences are predicted by MiPred to be real, the Pero3 sequence is predicted to be a false miRNA hairpin, and the Kangaroo rat sequence is predicted to be a pseudo miRNA. Note that the region with highest homology to known mir-467 family members in the Kangaroo rat is too close to the Drosha base.

The observation that the Old World rodent *Sfmbt2 *gene is imprinted, while other mammalian genes are not, prompted us to investigate the gene in other rodents. Rabbits, guinea pigs, squirrels and kangaroo rats are represented in the genome database. A BLAST search of intron 10 sequences against MirBase revealed that only kangaroo rats possessed a single potential mir-467 family miRNA sequence that folds appropriately with RNAFold (Figure 7). In the rodent phylogeny, kangaroo rats are within the mouse-related clade, although at a distance from *Mus *and *Rattus *[10]. *Peromyscus*, or deer mice, are indigenous to North America, and are evolutionarily more distant from Old World rodents (approximately 24 Mya), than rats and mice are from each other (approximately 12 Mya), but are closer than the other rodents with genomic sequence in the public database. They are represented by several species that occupy separate ranges, with clearly defined hybrid zones, where interbreeding is limited. There is evidence that some of the hybrid dysgenesis may be a function of incompatible genomic imprinting [11,12]. The *Peromyscus *genome is represented in the public database by 1.4 fold coverage of unassembled shotgun sequence traces. We used mouse exonic plus flanking intronic sequences to BLAST search the *Peromyscus *sequence database, and were able to assemble most of the *Sfmbt2 *coding sequence by analysing cDNA derived from *P. maniculatus *placenta. Primers from the exons flanking intron 10 were used to amplify a 10 kb sequence from *P. maniculatus *genomic DNA. Sequence analysis of this intron revealed the presence of three potential mir-467 sequences with good folding properties (Figure 7), although none of the putative pre-miRNAs resembled mouse or rat mir-467 outside of the mature miRNA sequence.

**Figure 8:**
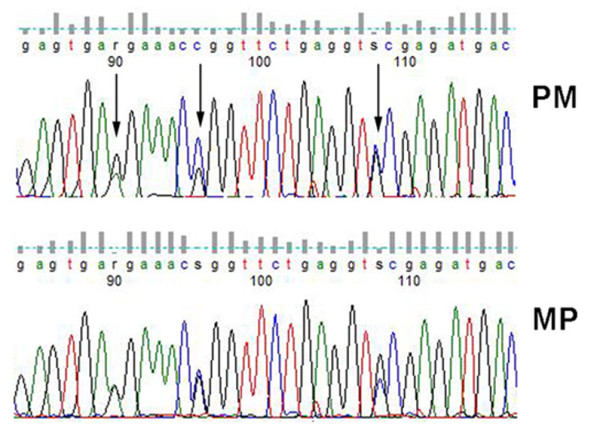
**Peromyscus *Sfmbt2 *is Biallelic**. *Peromyscus *placentas from interspecies crosses of *P. maniculatus *(M) and *P. polionotus *(P) display biallelic expression at several SNPs, indicating that the gene is not imprinted in either sub-species.

We next examined the allelic expression of *Sfmbt2 *in *Peromyscus *placenta. Analysis of placenta cDNA from reciprocal crosses of two sister species, *P. polionotus *and *P. maniculatus*, revealed that the *Peromyscus Sfmbt2 *gene is not imprinted in either species of deer mice (Figure 8).

## Discussion

The *Sfmbt2 *gene represented an entrée into a new imprinted domain, whose extent was unknown. Our analysis has revealed that it comprises a single coding gene, with a spliced antisense transcript that is transcribed from the first common intron and is also imprinted; this latter is likely a lincRNA [9], and its imprinted expression may reflect open/closed chromatin states of the parental alleles. No other genes tested within 4.3 Mb of *Sfmbt2 *display monoalleic expression in placenta, and published data from another study indicates that no genes in this domain are imprinted in e9.5 somatic tissues [5]. A recent computational analysis supported placental imprinting of *Sfmbt2*, using criteria heavily dependent on the two histone marks, H3K4Me3 and H3K27Me3, mentioned above [13]. No other genes within the domain examined in our study passed the computational test in this study, although one could argue that the criteria chosen for the machine learning exercise may have been biased.

The CpG island that spans the TSSs for the various *Sfmbt2 *transcripts is not likely to be regulated by DNA methylation since very little methylation exists at this locus in both placental and somatic tissues as measured by bisulfite sequencing. Only seven CpG sites (out of 140) display consistent methylation in oocyte genomic DNA. The four maternal CpGs from CG3 that were queried in placental DNA did not remain methylated, suggesting that DNA methylation is a secondary consequence of silencing mediated by some other mechanism. Indeed, methylation was largely asymmetric; of 25 cytosines that survived bisulfite mutagenesis in oocyte genomic DNA, 18 were asymmetric, and four of these resided within the context of a CpG dinucleotide, raising a question about whether reports of CpG methylation based on sequence analysis of only one strand are indeed symmetric as is assumed by most investigators. A recent report of extraembryonic tissue development in the absence of DNMT1, 3A and 3B supports the notion that DNA methylation has little if any function in placenta and yolk sac [14]. It is possible that asymmetric methylation of oocyte genomic DNA, perhaps mediated by DNMT3A/DNMT3L/DNMT3B, may drive establishment of a heritable chromatin structure that does not depend on continued DNA methylation.

There are three additional CpG islands within and near *Sfmbt2*; one in intron 11, one in intron 14 and another between *Sfmbt2 *and the next telomeric gene *Prkcq *(which is not expressed in extraembryonic tissues). None of these CpG islands is conserved between rats and mice, the intron 11 CpG island is methylated on both alleles in mouse placenta (not shown), and the other two CpG islands, which are part of a recently expanded retrotransposon family, are not present in *Mus castaneus *(not shown). The TSS CpG island is therefore the most likely regulatory region for *Sfmbt2*.

Single gene imprinted domains comprise a small subset of imprinted genes. Of six reported genes (*Gatm, Nnat, Nap1l5, Inpp5f_v2, Htr2a *and *Slc38a4*), five have well documented methylation analyses (*Gatm, Nnat, Nap1l5, Inpp5f_v2 *and *Slc38a4*). Four of these display methylation of the silenced (maternal) TSS [15-17], while no DMR could be found near *Gatm *[18], similar to our observations for *Sfmbt2*.

The Broad Institute ChIP-seq database indicates that there is very strong association of the CpG island with Ring1b, EZH2, SUZ12, H3K4 Me2/3 and H3K27Me3, but not H3K9 Me2 or Me3, nor H4K20Me3 in ES cells. Interestingly, the human gene also displays high H3K4Me3 and H3K27Me3 binding, and measurable although reduced EZH2 and Ring1b association in ES cells. Moreover, the human gene is associated with two antisense lincRNAs, one at each end of the *SFMBT2 *primary transcript. This is interesting because the human gene is not imprinted. Some other aspect of chromatin structure may be important for imprinted regulation of *Sfmbt2 *in extraembryonic tissues.

Rodents from the *Mus *and *Rattus *genuses imprint their *Sfmbt2 *gene, while other mammals do not, including rodents from the Peromyscine genus. The acquisition of a large block of miRNAs within an intron of the rodent gene strongly suggests that some aspect of miRNA biology is driving silencing of this locus from the maternal allele in early embryos and extraembryonic tissues. A survey of miRBase reveals that there are approximately 370 miRNA loci, some of which are clusters, in the mouse genome, which is roughly equivalent to 1.5% of protein coding genes. Of 96 imprinted genes, 11 (12.5%) contain miRNAs, while 20% contain some kind of small ncRNA including miRNAs; 84% of these gene arrangements are conserved in humans. The numbers represent a significant enrichment among imprinted genes for co-regulation by some kind of ncRNA. One could speculate that local expansion of ncRNAs might flag a gene for silencing in one of the parental germlines, and that selection would fix the silencing, perhaps by insertion of a more efficient DMR, thus obviating the need for retention of the ncRNA cluster.

The annotated miRNAs in both the rat and mouse arrays are from the mir-467 family. However, it is clear that there are likely more miRNAs in each block than have been annotated. Dot plots of mouse and rat introns against both themselves and each other reveal significant repetitive sequence homology, even after the annotated miRNAs are removed (Additional file 3, Figure S3). In addition, both mouse and rat arrays can be organized into tandem repeats of approximately 2 kb (Additional file 4, Figure S4), although the rat repeats suffer from gaps in the sequence database. In contrast, no other mammalian introns examined contain repetitive sequences. There may be more miRNAs, or at least miRNA-like sequences, in both mouse and rat introns that remain to be annotated. For example, a small random section of the rat intron was subjected to analysis with MiPred [19] to reveal the presence of two additional predicted pre-miRNA sequences from the mir-467 family (Additional file 5, Figure S5).

Kangaroo rats and deer mice fall within the mouse related clade of rodents [10]. Both possess potential mir-467 miRNA sequences in intron 10, although these do not resemble the mir-467 pre-miRNA sequences in rats and mice, apart from the putative mature miRNA. It is unclear whether these potential miRNAs in kangaroo rats and *Peromyscus *are evolutionary precursors or remnants.

Several of the miRNAs from the murine *Sfmbt2 *locus are expressed and processed in placenta (Additional file 6, Figure S6), not surprising given that they are part of the primary transcript for *Sfmbt2*, which is robustly expressed in placenta. Recent analysis of small RNA libraries with massively parallel sequencing strategies have indicated that several of the miRNAs from the cluster are expressed in ES cells [20]; interestingly, the number of reads does not correlate with the frequency of the miRNA within the block of repeats, suggesting that the primary transcript likely folds in a complex manner that only allows processing of a subset of the potential miRNAs. Whether these miRNAs mediate any functions in placental biology remains an open question. There are mir-467 family members in other mammalian genomes, notably human and pig, although in far fewer numbers than in rodents. Moreover, there are several mmu-mir-467 at other locations in the mouse genome. Any of the miRNAs processed from the *Sfmbt2 *primary transcript would be imprinted, although testing this is problematical given the highly repetitive nature of the array itself, and the presence of other family members at other locations in the genome.

Genomic imprinting is an epigenetic mechanism that is restricted to mammals and angiosperm plants. There is mounting evidence that it is mediated by maternal factors, possibly as a means of controlling extraembryonic tissues that parasitize the maternal reproductive tract [21]. Studies of the evolution of imprinting indicate that it seems to have emerged at different times at different loci [22-24]. Interestingly, several imprinted clusters contain miRNAs or SNORDs, and some of these have been demonstrated to be imprinted themselves. The role of ncRNAs in imprinting also includes regulatory functions, such as that exerted by the *Kcnq1ot1 *antisense transcript at the *Kcnq1 *locus, and the *Airn *transcript at the *Igf2r *locus [6].

RNAi biology is providing interesting new insights into gene regulation. Studies in plants and yeast have revealed a wide array of effects, from post transcriptional gene silencing involving both mRNA degradation and translational control, to heterochromatin formation, particularly at centromeres. A recent report of the ncRNA clusters at the *Dlk1 *and *Snrpn *imprinted loci has revealed that transcripts containing precursors of the miRNAs or SNORDs from these loci are retained by the nucleus [25]. This raises the interesting notion that the function of the ncRNAs at these loci may be more similar to the regulation of centromeres in yeast than to "traditional" cytoplasmic RNAi. Such a role is also implied by the phenotype of both *Dicer *and *Ago2 *conditional null alleles in mouse oocytes, which fail to complete the first meiotic division due to misaligned chromosomes [26,27]. Interestingly, maternal loss of *Dgcr8*, a component of the miRNA processing pathway, is without effect on development, even though the profile of misexpressed miRNAs in maternal *Dicer *-/- and *Dgcr8 *-/- zygotes is the same [28]. It is tempting to speculate that expression of a subset of the miRNAs from the *Sfmbt2 *cluster provides the RNAi transcriptional silencing biochemistry with the molecular substrates that allow it to target the repeated sequences at the *Sfmbt2 *intron for transcriptional epigenetic silencing on the maternal allele.

The evolution of miRNAs is highly dynamic, and is characterized by expansions and contractions, partly through segmental duplication/deletion events. The mir-467 family was acquired by amniotes approximately 350 Mya [29], and has remained relatively small; humans possess one copy on Chromosome 3, and another on the X chromosome, while pigs and orangutans possess only the X linked gene. Mice have acquired, in addition to the large cluster at *Sfmbt2*, several other copies on Chromosomes 5, 9, 10, 11 and 13, although current miRNA annotation and archiving may be incomplete for this family. Interestingly, a large cluster of miRNAs in a different family expanded in primates after divergence from the rodent lineage, and is expressed exclusively in placenta [30]. This cluster maps to Chromosome 19q13.4, and is near several loci that display either imprinted expression or parent of origin effects in humans (see imprinted gene catalogue records http://igc.otago.ac.nz/Search.html). A recent report demonstrated paternal expression of the primary transcript encoding this miRNA cluster in human placenta [31]. The primary transcript is associated with a classic germline DMR; this locus is however much older than the cluster at the rodent *Sfmbt2 *gene by at least 30 million years. As mentioned above, two other imprinted loci, the *Dlk1 *domain and the *Snrpn *region, also contain large clusters of non-coding RNAs (miRNAs and SNORD genes). They also possess classic germline DMRs. The lack of any noticeable germline methylation at *Sfmbt2 *may reflect its youth as an imprinted domain, suggesting that imprinted regulation precedes establishment of differential methylation. This raises the possibility that one general mechanism by which genes or gene domains become imprinted is through local expansion of miRNAs [32], and that the rodent *Sfmbt2 *locus is a window on an actively evolving imprinted domain. How these clusters of ncRNAs mediate gene silencing at the transcriptional level will be an interesting puzzle to solve.

## Conclusions

The very recent insertion of a large block of miRNAs into the intron of the PcG gene *Sfmbt2* in old world rodents coincides with the acquisition of placental imprinting at this locus. The transcriptional start site CGI does not display heritable differential methylation, suggesting that maternal silencing by other chromatin mechanisms mediates imprinting at this locus.

## Methods

### Animals

Mice and rats were bred using standard animal husbandry. Timed pregnancies were assessed by the appearance of a vaginal plug on day 0.5 in mice, and by the presence of a copulatory plug in the bottom of the cage on day 0.5 in rats. *Peromyscus *(PO - *P. polionotus *and BW - *P. maniculatus *stocks) were obtained from the breeding colonies of the *Peromyscus *Genetic Stock Center. Reciprocal interspecific hybrids were produced by pairing PO with BW animals. *Peromyscus *pregnancies were assessed visually. Bovine blastocysts were produced by *in vitro *fertilization of domestic cattle oocytes matured from follicles obtained from slaughterhouse ovaries with frozen-thawed plains bison sperm stored in liquid nitrogen at the Toronto Zoo. Bovine embryos were cultured in modified synthetic oviductal fluid medium supplemented with 2% steer serum until the expanded blastocyst stage before extraction of RNA from pools of five embryos. All procedures involving laboratory animals were approved by the Canadian Council on Animal Care, by a licence from the UK Government Home Office (80/2042), or by the Guide for the Care and Use of Laboratory Animals, USA.

### Human placenta

This study was conducted according to the principles expressed in the Declaration of Helsinki. Informed consent was obtained from each individual patient and tissue collections were approved by the Mount Sinai Hospital's Review Committee on the Use of Human Subjects and carried out in accordance with the participating institutions' ethics guidelines. First and second trimester human placental tissues were obtained immediately following elective termination of pregnancies by dilatation and curettage, or suction evacuation. Gestational age was determined by the date of the last menstrual period and first trimester ultrasound measurement of crown-rump-length (CRL). Term placental tissue was collected at the time of delivery from healthy pregnancies with normally grown fetuses that did not have signs of placental dysfunction. Samples were collected randomly from central and peripheral placental areas and snap frozen immediately after delivery. Calcified, necrotic and visually ischemic areas were excluded from collection.

### cDNA Synthesis

Total RNA isolated with Trizol reagent according to manufacturer's instructions was used as template for the synthesis of cDNA, using random hexamers as primers and either Superscript III (Invitrogen) or RevertAid (Fermentas) cDNA synthesis kits. All cDNA synthesis reactions were preceded by a DNAse I step to eliminate any contaminating genomic DNA; an aliquot of the DNAse treated RNA was set aside as a zero R.T control. No PCR product was ever obtained with the zero R.T control samples. PCR was performed with either LA Taq (Takara) or Bioline Taq (Bioline International).

### Peromyscus *Sfmbt2* cDNA and Genomic Sequence

BLAST analysis of sequence trace files for *Peromyscus maniculatus *using murine exon sequence plus some flanking intron sequence as search query yielded fourteen coding exons with strong homology. The seven missing exons were recovered with primers designed from the *Peromyscus *sequence derived either from the database or from first pass sequence analysis of *Sfmbt2 *cDNA (Additional file 7, Table S2). The *Peromyscus Sfmbt2 *cDNA sequence accession number is [Genbank:HQ416417].

For analysis of *Peromyscus *intron 10, forward and reverse primers were designed from the exonic sequences of exons 9 and 10, respectively for amplification of genomic DNA. PCR product was sequenced with a series of primers in a walk from each end until the amplifiable intron sequence was small enough to clone into pT-Easy. Subclones of the pT-Easy intron fragments were sequenced with universal primers, and the full length intron 10 sequence was assembled. Some of the intron sequence was obtained from the sequence trace files. The *Peromyscus *intron 10 genomic DNA sequence accession number is [Genbank:HQ416418}.

### SNP analsysis

Annotated SNPs were chosen for analysis; where possible, SNPs that created RFLPs were selected. Primers were designed to amplify the selected SNPs, and PCR product was subjected either to restriction enzyme digestion or direct sequence analysis. Primers and analysis strategy are listed in Additional file 7, Tables S1 and S2. Where no SNPs could be found in the public database, genomic DNA from parental species was surveyed by sequence analysis.

### Bisulfite Mutagenesis

Genomic DNA was treated with sodium bisulfite, using a MethylCode kit from Invitrogen. For the isolation of oocyte genomic DNA, 10 - 20 females aged 5-10 weeks were superovulated with 5 I.U. of PMS administered intraperitoneally followed 48 hours later by 5 I.U. of hCG. Eighteen hours after the hCG injection, oocytes were retrieved from the oviducts and stripped of cumulus cells with a combination of hyaluronidase treatment, vigourous pipetting, and removal of the zona pellucida with acidic tyrodes. This latter step ensured that all cumulus cells were removed from the sample before extraction of genomic DNA, which involved a 5 hour treatment with Proteinase K followed by ethanol precipitation.

With the exception of CG3 top strand, bisulfite primers were designed with the aid of the MethPrimer web based tool (http://www.urogene.org/methprimer/index1.html) (Additional file 7, Table S3). Only one strand was sequenced for amplicons Me3, CG1, (bottom strands), and Me4, CG2 (top strands). CG3 was sequenced from both strands. The primers used for amplification of the top strand were manually designed to avoid bias generated by asymmetric methylation. Oocyte genomic DNA required further amplification with nested primers, so all samples were subjected to nested amplification prior to cloning into pGEM-TEasy, using a kit from Promega. Plasmid DNA was purified from individual clones and subjected to sequence analysis with the T7 universal primer. Occasional samples were sequenced with the SP6 universal primer. Only unique clones were reported; uniqueness was assessed by random PCR generated SNPs, some of which were the result of less than complete bisulfite mutagenesis. Only clones exhibiting 95% mutagenesis of non-CG cytosines were scored.

## Abbreviations

Mb: megabase; e7.5: embryonic day 7.5; miRNA: micro RNA; kb: kilobase; DMR: differentially methylated region; ChIP-seq: chromatin immunoprecipitation massively parallel sequence; H3K4Me3: histone H3 lysine 4 trimethylation; H3K27Me3: histone H3 lysine 27 trimethylation; H3K9Me3: histone H3 lysine 9 trimethylation; H4K20Me3: histone H4 lisine 20 trimethylation; ES: embryonic stem cells; 3'UTR: 3' untranslated region; Mya: million years ago; lincRNA: long intergenic non coding RNA; DNMT: DNA methyltransferase; ncRNA: non coding RNA; TSS: transcriptional start site

## Authors' contributions

All of the molecular analysis was performed by QW, JC, JH, JT, EDC, KM and SV. Some of the work was performed in the lab of AFS while SV was on sabbatical leave. Bovine embryo cDNA was provided by PS and GM. Peromyscus tissues were provided by PV. Human cDNA placental cDNA was provided by IC. Rat genomic sequence data were provided by CM. The manuscript was written by SV, with input from all authors; all authors approved the final manuscript.

## Supplementary Material

Additional file 1 **Figure S1 Human *SFMBT2 *is Biallelic**. Placenta RNA from four individuals (#6 - 18 weeks gestation; #8 - 39 weeks gestation; #9 - 40 weeks gestation; #10 - 41 weeks gestation) was converted to cDNA as described in Materials and Methods, and amplified with primers for two SNPs found at high frequencies in the human population (see Primer Table for details). Two samples amplified successfully with SNP1 primers, and three with SNP3 primers. In both cases, alleles were identified by digestion with diagnostic restriction enzymes; asterisks (*) indicate one allele and number sign (#) indicates the other allele. Sample #6 was homozygous for both SNPs. The other samples contained two alleles, indicating the presence of both maternal and paternal alleles. Click here for file

Additional file 2 **Figure S2 Allelic Analysis of Genes in the Murine *Sfmbt2 *Domain**. Placenta cDNA from e14.5 (C57BL6 X Castaneus) F1 embryos was analysed either by direct sequencing of PCR product, or by restriction enzyme digestion with the indicated enzymes. Click here for file

Additional file 3 **Figure S3 Dot Plot Comparison of Rat and Mouse Intron 10**. Sequence from rat and mouse Sfmbt2 intron 10 was plotted against each other or against itself. Note the large segment of highly repetitive sequence, even after the removal of annotated miRNA sequence. In contrast, human, kangaroo rat and cow introns generate the predicted diagonal line when self plotted. Click here for file

Additional file 4 **Figure S4 Alignment of Repeats in Mouse and Rat Intron 10**. The block of miRNAs is arranged in a series of tandem repeats of an approximately 2.5 kb sequence, each of which contains 6-7 miRNAs. The mouse repeats align well with each other, while the rat repeats are less well conserved. Click here for file

Additional file 5 **Figure S5 Annotation of miRNAs May Be Incomplete**. A small segment of the rat intron close to an annotated miRNA (yellow highlighting), when subjected to MiPred reveals the presence of two additional potential mir-467 family members (green highlighting) that fold into good potential pre-miRNAs with RNAFold (http://rna.tbi.univie.ac.at/cgi-bin/RNAfold.cgi). Click here for file

Additional file 6 **Figure S6 MiRNAs from the Intron 10 Cluster Are Expressed in Placenta**. Five of the most commonly annotated miRNAs from the Sfmbt2 intron 10 cluster were assayed by RT-PCR in placenta RNA. The asterisk indicates a primer dimer artifact. Click here for file

Additional file 7 **Tables S1-S4**. Tables 1-4 list primer sequences used for analyses described in the paper. Click here for file
