# Supplementary figures and images for: Recent acquisition of imprinting at the rodent Sfmbt2 locus correlates with insertion of a large block of miRNAs
Source: BMC Genomics. 2011 Apr 21;12:204. doi: 10.1186/1471-2164-12-204 (PMC3110154; doi:10.1186/1471-2164-12-204)

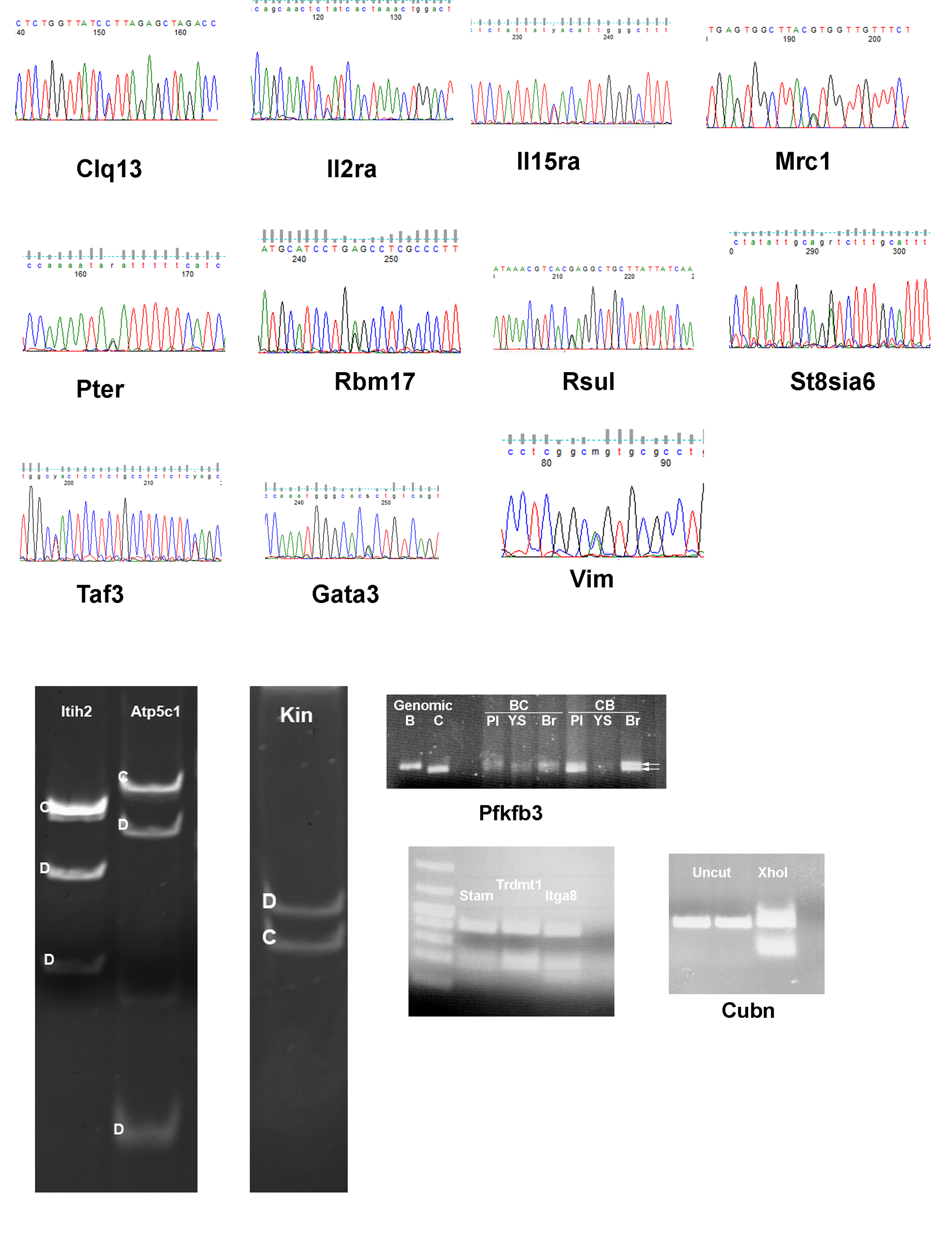

Supplement: Additional file 1 — Figure S1 Human SFMBT2 is Biallelic. Placenta RNA from four individuals (#6 - 18 weeks gestation; #8 - 39 weeks gestation; #9 - 40 weeks gestation; #10 - 41 weeks gestation) was converted to cDNA as described in Materials and Methods, and amplified with primers for two SNPs found at high frequencies in the human population (see Primer Table for details). Two samples amplified successfully with SNP1 primers, and three with SNP3 primers. In both cases, alleles were identified by digestion with diagnostic restriction enzymes; asterisks (*) indicate one allele and number sign (#) indicates the other allele. Sample #6 was homozygous for both SNPs. The other samples contained two alleles, indicating the presence of both maternal and paternal alleles. [file 1471-2164-12-204-S1.TIFF]

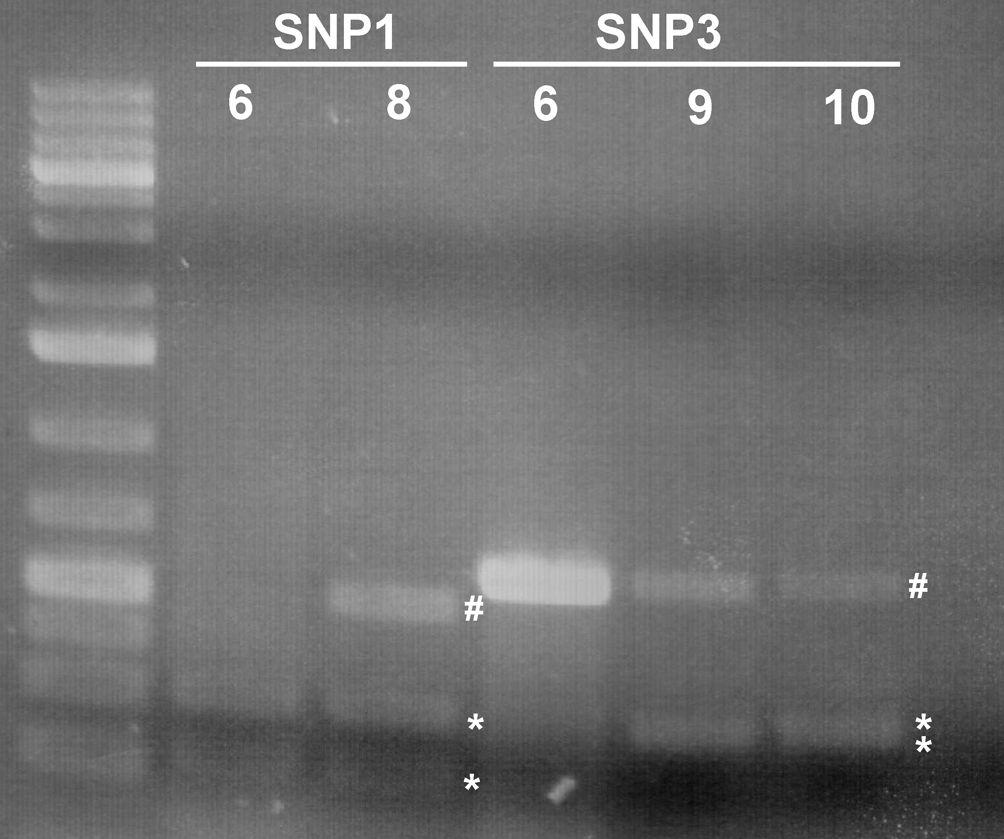

Supplement: Additional file 2 — Figure S2 Allelic Analysis of Genes in the Murine Sfmbt2 Domain. Placenta cDNA from e14.5 (C57BL6 X Castaneus) F1 embryos was analysed either by direct sequencing of PCR product, or by restriction enzyme digestion with the indicated enzymes. [file 1471-2164-12-204-S2.TIFF]

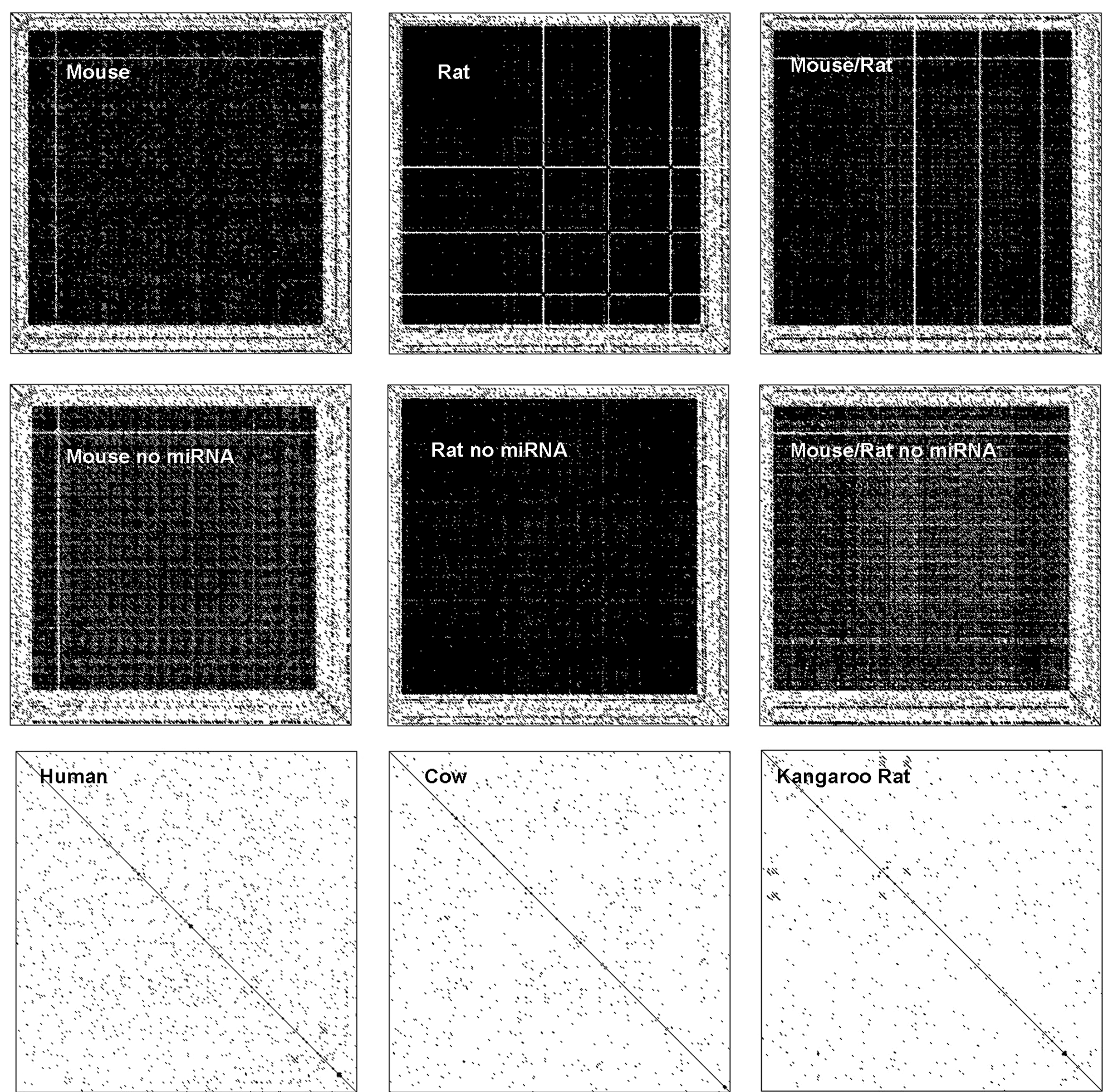

Supplement: Additional file 3 — Figure S3 Dot Plot Comparison of Rat and Mouse Intron 10. Sequence from rat and mouse Sfmbt2 intron 10 was plotted against each other or against itself. Note the large segment of highly repetitive sequence, even after the removal of annotated miRNA sequence. In contrast, human, kangaroo rat and cow introns generate the predicted diagonal line when self plotted. [file 1471-2164-12-204-S3.TIFF]

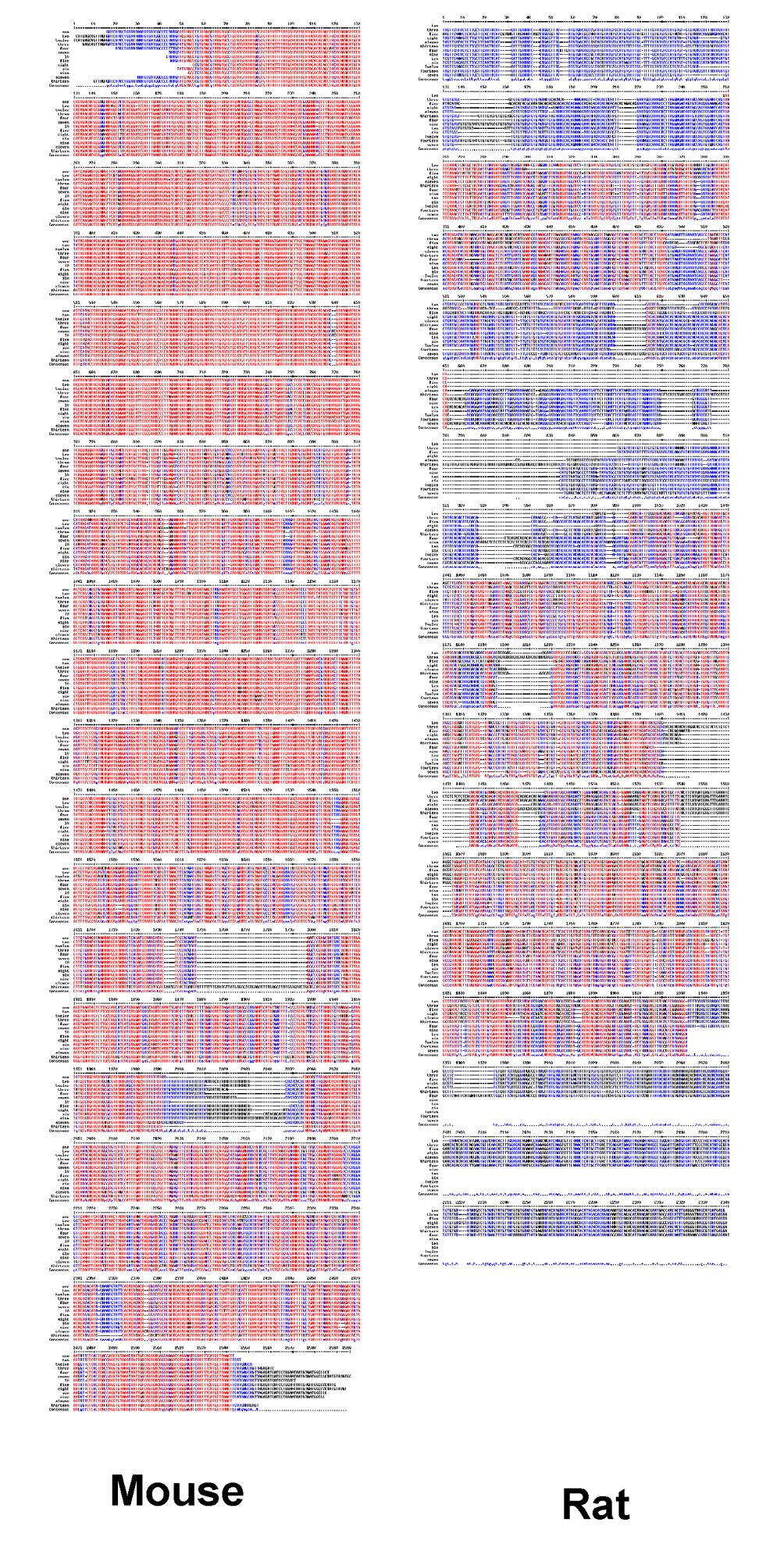

Supplement: Additional file 4 — Figure S4 Alignment of Repeats in Mouse and Rat Intron 10. The block of miRNAs is arranged in a series of tandem repeats of an approximately 2.5 kb sequence, each of which contains 6-7 miRNAs. The mouse repeats align well with each other, while the rat repeats are less well conserved. [file 1471-2164-12-204-S4.TIFF]

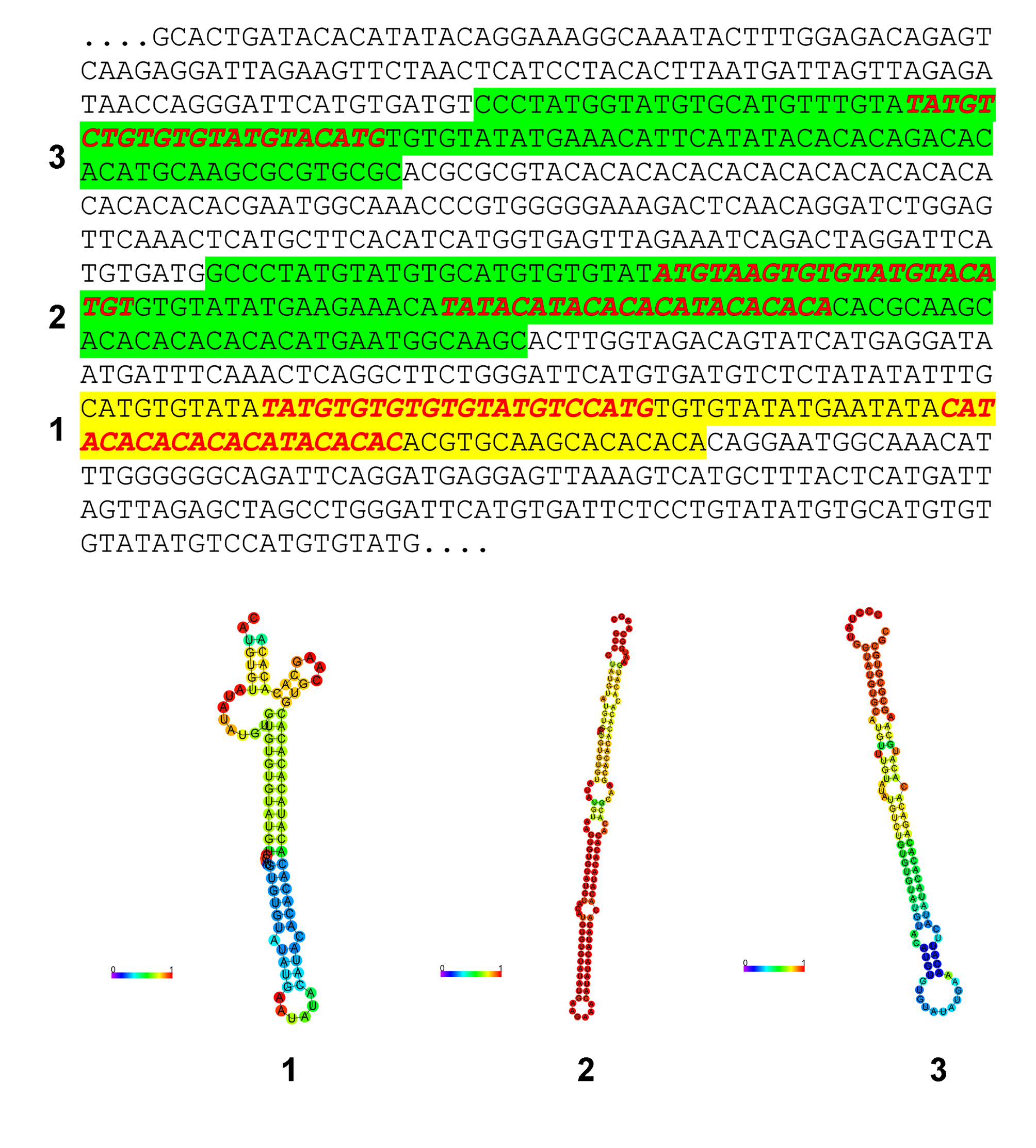

Supplement: Additional file 5 — Figure S5 Annotation of miRNAs May Be Incomplete. A small segment of the rat intron close to an annotated miRNA (yellow highlighting), when subjected to MiPred reveals the presence of two additional potential mir-467 family members (green highlighting) that fold into good potential pre-miRNAs with RNAFold (http://rna.tbi.univie.ac.at/cgi-bin/RNAfold.cgi). [file 1471-2164-12-204-S5.TIFF]

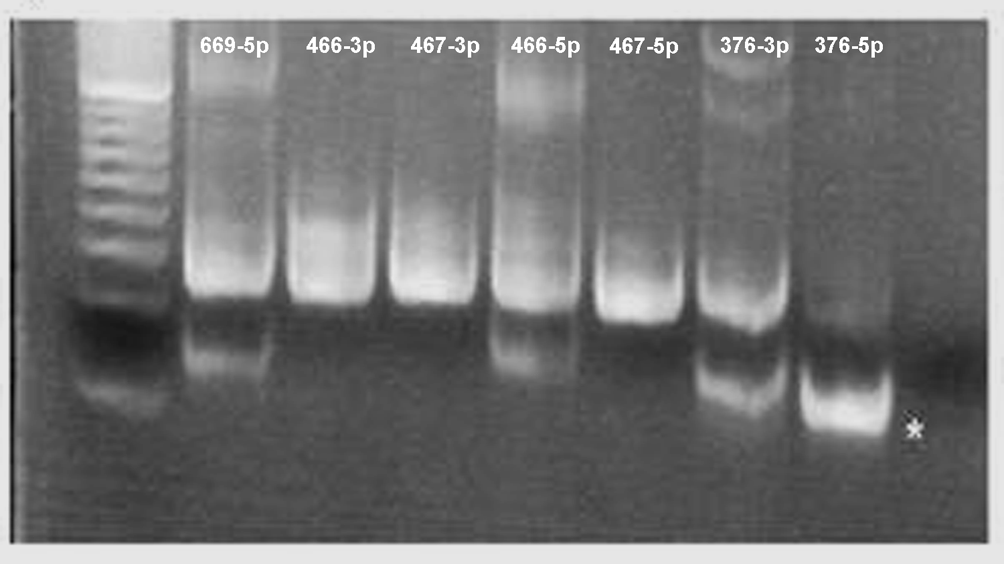

Supplement: Additional file 6 — Figure S6 MiRNAs from the Intron 10 Cluster Are Expressed in Placenta. Five of the most commonly annotated miRNAs from the Sfmbt2 intron 10 cluster were assayed by RT-PCR in placenta RNA. The asterisk indicates a primer dimer artifact. [file 1471-2164-12-204-S6.TIFF]
