# Supplementary material for: Recent acquisition of imprinting at the rodent Sfmbt2 locus correlates with insertion of a large block of miRNAs
Source: BMC Genomics. 2011 Apr 21;12:204. doi: 10.1186/1471-2164-12-204 (PMC3110154; doi:10.1186/1471-2164-12-204)
Supplement: Additional file 7 — Tables S1-S4. Tables 1-4 list primer sequences used for analyses described in the paper. [file 1471-2164-12-204-S7.DOC]

Table S1 Primers Used For Domain Gene SNP Analysis

| Gene | Forward | Reverse | SNP Sequence | RFLP |
| --- | --- | --- | --- | --- |
| Ankrd16 | GTCAGCATGTGGCTTGCAC | CTGGTGATGTGCTTCAGTGG |  | SsiI |
| Atp5c | TGCTGCAGAATTACCAGGAG | AAGATAATTTACAAACATCCTGCTGTC |  | HhaI |
| C1ql3 | TTGGTGTCCTGTGATCTTGC | TTCACACTTAGAGGGGAATCCT | TTGGTGTCCTGTGATCTTGC |  |
| Cubn | AAACCAAGTGACTCAGTTCTGC | TCCATGGTGGGTGTCTGAGT |  | XhoI |
| Fbxo18 | ACGCTGAGGGTGACATGATT | CAACATTCCAAGAAGCCCATA |  | HhaI |
| Gata3 | CCATGGGTTAGAGAGGCAGA | CACACAGGGGCTAACAGTCA | TCTGCATTCCAACCACTGAA |  |
| Il2ra | CCGCTCCATCTTCCTCTTCT | TTTCCAGTGTTTTCACGGTATG | CCGCTCCATCTTCCTCTTCT |  |
| Il15ra | CGTGTTGAGGTGGAAACCAT | CAATGGGAGCCTTGTCAATC | CGTGTTGAGGTGGAAACCAT |  |
| Itga8 | ACAATGGAAACCCAAGAGGT | CGTTTGATGGCTCCTTTTTG |  | HpaII |
| Itih2 | CGACACAGCTCATCTTGGAA | GGTGACAGTCAGCTTATGTCCTT |  | HaeIII |
| Itih5 | GAAGAAAGGAATAGAAATGGGTCA | GTAGACCAGGTTGGCCTCAG |  | EcoRV |
| Kin | GGACAGACGCCTGGTTACAG | TTTAATACAAAACAAATCCCCTAACA |  | HpaI |
| Mrc | TCCTGGCAAGATTAGAGAAACA | GCCTGGGACAAACAAGATTC | TCCTGGCAAGATTAGAGAAACA |  |
| Pfkfb3 | CCAAAGGACCCTTTATGACG | TGGTGGTGGCTCATGCTTAT |  | size |
| Pter | CCCTGAGACACTATCAGGAATG | TGCTCCACTGACATGGCTCTGGT | CCCTGAGACACTATCAGGAATG |  |
| Rbm17 | CAGTGGGTTTTCAAGACGAC | CTCCCATTCAGATCCACCAC | CAGTGGGTTTTCAAGACGAC |  |
| RsuI | TGCTGGTGCCTACTTTTCTTG | TTTTGTCAAGAAGAAACGTGTGAT | TGCTGGTGCCTACTTTTCTTG |  |
| Sfmbt2 AS | CCTGAGGGAAAATTCAGAAG | AGATGACTTGCCAAGAGAGGTC | CCTGAGGGAAAATTCAGAAG |  |
| St8sia6 | CTCCTCCTGCGTATGCTCTG | CTCATGTTAGTCCCCACTGGA | CTCCTCCTGCGTATGCTCTG |  |
| Stam | GCTCTCGTAGCCAAGGACCCTG | TCTATCTGAACATCATCATTGAACTG |  | HhaI |
| Taf3 | ATTGAAATGCAGCCGAAAAAGC | TGTGTAGAGGCTCAGGAGTGG | ATTGAAATGCAGCCGAAAAAGC |  |
| Trdmt1 | TGCTGAAAGACTTCCTTGAAGA | CAGGACTGTGAGGAGTTTTGC |  | HhaI |
| Vim | TACCAGGTCTGTGTCCTCGTC | GCTGCTCGAGCTCAGCCAGC | TACCAGGTCTGTGTCCTCGTC |  |

Table S2 Primers Used For Analysis of Human, Rat, Peromyscus and Bovine Sfmbt2, and for sequencing of Peromyscus intron 10 (Pint)

| Amplicon | Forward | Reverse | SNP Sequence | RFLP |
| --- | --- | --- | --- | --- |
| Human SNP1 | GGAGCTTGTTTCAGCTCCTC | GAAAGGGGTGTTTGGCATAA |  | BstBI |
| Human SNP3 | CCCTTTCACATGGTTGAACTC | CACAAGATAGGAATCCAATTTCA |  | BsiEI |
| Rat SNP | AGGAGGCTGCTTTAGGGAAG | CAAGCAACCCCAAAAGAG |  | SsiI |
| Cow SNP outer | AGTGTCACGTGGCGTTCAT | GGGGTCATGCCTTCCTTACT |  |  |
| Cow SNP nested | GTCTCTGTAGGGTTCACCGCT | GTTGACGGAAGCGTCCTTAG | GTCTCTGTAGGGTTCACCGCT |  |
| Pero Ex1-Ex7 | GAGACGTTTCCCAGTTTCCA | TCTCCAGCTTCATCCCAACT |  |  |
| Pero Ex5-Ex14 | CCAGTTGGTTGGTGTCAAGA | GCAATCCTCCCTTTGTTCAG |  |  |
| Pero Ex13-Ex19 | CCACAGTGCCTGTTGAGAAA | AACTGTTCCGCAGGGTCAC |  |  |
| PeroSNP | CGTCGGAAATCCATCTTTGT | TCTCCCTCAACAGCAGAGGT | CGTCGGAAATCCATCTTTGT |  |
| Pint1 | CCCGAACCTAACAAACTCTCC | CAGAAGGGTGGTGCTTCTTC |  |  |
| Pint2 | GGGTTTTTGTTGGCTACCTG | TGGCAAAGGAAGTTGGTAGC |  |  |
| Pint3 | TCTGGGCATTTCTGTGTGAG | CTCCCCTTCCTTTCAAGACC |  |  |
| Pint4 | TGTGTTCACCTGTGTGCAAG | GGGAAGGAGGACAGGAGAAA |  |  |
| Pint5 | TGAAACATGCCTTTCAGCAG | TGCCACCTATGTTTGGTTTTG |  |  |
| Pint6 | TAAAGAGCCAGTGGTGACCTG | CAAATGGTATGGGTGCAAGG |  |  |
| Pint7 | AGTTTGATGCCTTTTCTTTCTGTC | TGTGAAAGGATTTCAGCAATAAAA |  |  |

Table S3 Bisulfite Primers

| Amplicon | Location | Forward (outer) | Reverse (outer) | Forward (nested) | Reverse (nested) |
| --- | --- | --- | --- | --- | --- |
| Me3 (bot) | 10292101 – 10292602 | AAGATTGAGTTTTGTTAGTTTGAGAATATT | ATAACTTTAAACCTCTTTCCCACCT | TTGTTAGTTTGAGAATATTTTTTTTATA | CACCTCTTTCTAAAAAATCAACTAC |
| Me4 (top) | 10293003 – 10293596 | GGGTTGTAAAGGTAAAGATTTTGAGTA | AAAAAAAATAAAAAACCCTAAAAACAAA | GTAATTGTGTTTTTTGTTTTTTAGT | AATCTACCCCACCCTATACTTTAATTATAC |
| CG1 (bot) | 10294078 – 10294783 | ATGTATATTAAAAGAGGGAGATTT | AAATTCCACCATTCCTAACAAAAC | ATGTATATTAAAAGAGGGAGATTT | CCATTCCTAACAAAACAAACTTAATC |
| CG2 (top) | 10295210 – 10295934 | AGAGTGTAGTAAAATAAGGGTTAATTATTA | CCCCAACCCAATCAATACTTATAT | AATTATTAAATGTGTTTTAGATTTAATTT | CCCCAACCCAATCAATACTTATAT |
| CG3 (bot) | 10296104 – 10297003 | GGAATTATGGTTGAAATTGTAGTTTATAAA | ACCAACTAACCTAAACCTAAAAAAA | TATTTTGTGTTTGTTTTTATTTTAATAGAA | ACCAACTAACCTAAACCTAAAAAAA |
| CG3 (top) | 10296036 - 10297241 | GTATGGGGAGGTYTTGAAGTGGAATT | TACTACACTCCAARTGCCCCAAATCCCCTCT | GTATGGGGAGGTYTTGAAGTGGAATT | CACTCARCTCCTCCTCTTCCCACC |

NOTE: CG3 Top primers were designed to amplify both converted and non-converted DNA to avoid potential bias created by asymmetric methylation.

Table S4 miRNA RT-PCR Primers

| Primer name | Sequence |
| --- | --- |
| 669a-5p | AGTTGTGTGTGCATGTTCATGT |
| 466b-3p | TATACATACACGCACACATAAGA |
| 467a-3p | ATATACATACACACACCTACAC |
| 466b-5p | GATGTGTGTGTACATGTACATG |
| 467a-5p | TAAGTGCCTGCATGTATATGCG |
| 376a-3p | ATCGTAGAGGAAAATCCACGT |
| 376a-5p | GGTAGATTCTCCTTCTATGAGT |
| Anchor | GCGAGCACAGAATTAATACGACTCACTATAGGTTTTTTTTTTTTVN |
| Common reverse | GCGAGCACAGAATTAATACGACTCAC |
